# Analysis of the Performance of 11 Formulae for Fetal Weight Estimation in Preterm Fetuses with Abnormal Doppler Velocimetry – A Retrospective Multicenter Study

**DOI:** 10.1055/s-0038-1670643

**Published:** 2018-09-20

**Authors:** Alessandra Martins Heringer de Lima, Paulo Roberto Nassar de Carvalho, Saint Clair Gomes Junior, Ana Carolina Costa Carioca, José Maria de Andrade Lopes

**Affiliations:** 1 Diagnostic Center, Clínica Perinatal Laranjeiras, Rio de Janeiro, RJ, Brazil; 2 Fetal Medicine Center, Instituto Nacional Fernandes Figueira, Fundação Oswaldo Cruz, Rio de Janeiro, RJ, Brazil

**Keywords:** fetal weight, birth weight, premature birth, ultrasound, prenatal ultrasound, doppler ultrasound, fetal growth retardation, placental insufficiency, peso fetal, peso ao nascer, parto prematuro, ultrassonografia, ultrassonografia pré-natal, ultrassonografia doppler, retardo do crescimento fetal, insuficiência placentária

## Abstract

**Objective** To assess 11 formulae commonly used to estimate fetal weight in a population of premature fetuses who had abnormal Doppler velocimetry due to early-onset placental insufficiency. The performance of each formula was evaluated in subgroups of fetuses with expected growth and intrauterine growth restriction.

**Methods** Data were collected from fetuses and mothers who delivered at three Brazilian hospitals between November 2002 and December 2013. We used the following formulae: Campbell; Hadlock I, II, III, IV and V; Shepard; Warsof; Weiner I and II; and Woo III.

**Results** We analyzed 194 fetuses. Of these, 116 (59.8%) were considered appropriate for gestational age (AGA), and 103 (53.1%) were male. The amniotic fluid volume was reduced in 87 (44.8%) fetuses, and the umbilical artery Doppler revealed absence or inversion of diastolic flow in 122 (62.9%) cases, and the analysis of the ductus venosus revealed abnormal flow in 60 (34.8%) fetuses. The Hadlock formulae using three or four fetal biometric parameters had low absolute percentage error in the estimated fetal weight among preterm fetuses with abnormal Doppler studies who were born within 5 days of the ultrasound evaluation. The results were not influenced by the clinical and ultrasound parameters often found in early-onset placental insufficiency.

**Conclusion** In this study, the formulae with the best performance for fetal weight estimation in the analyzed population were Hadlock I and IV, which use four and three fetal biometric parameters respectively to estimate the weight of preterm fetuses with abnormal Doppler studies.

## Abstract (pt)

**Resumo:**

**Objetivo** Avaliar o desempenho de 11 fórmulas comumente utilizadas para estimativa de peso fetal em uma população de fetos prematuros com dopplervelocimetria alterada devido a insuficiência placentária de início precoce. O desempenho de cada fórmula foi avaliado em subgrupos de fetos com crescimento adequado e com crescimento intrauterino restrito.

**Métodos** Foram coletados os dados de mães e fetos cujos partos foram acompanhados em 3 instituições brasileiras entre novembro de 2002 e dezembro de 2013. As fórmulas selecionadas para análise foram: Campbell; Hadlock I, II, III, IV e V; Shepard; Warsof; Weiner I e II; e Woo III.

**Resultados** Foram analisados os pesos de 194 fetos, dos quais 116 (59,8%) foram considerados adequados para a idade gestacional, 103 (53,1%) eram do sexo masculino, em 87 (44,8%) havia redução do volume de líquido amniótico, em 122 (62,9%) o Doppler de artéria umbilical demonstrou ausência ou inversão do fluxo na diástole, e em 60 (34,8%) a análise do duto venoso indicou fluxo anormal. A média do erro percentual absoluto (EPA) demonstrou que as fórmulas de Hadlock que utilizam 3 ou 4 parâmetros biométricos fetais apresentaram o melhor desempenho. Os resultados obtidos para essas fórmulas não sofreram influência dos parâmetros clínicos e ultrassonográficos frequentemente encontrados na insuficiência placentária de início precoce.

**Conclusão** O presente estudo demonstrou o melhor desempenho das fórmulas de Hadlock que contêm 3 ou 4 parâmetros da biometria para estimativa de peso de fetos prematuros com anormalidades ao mapeamento Doppler.

## Introduction

The accuracy of fetal weight estimation is very important for patients who have intrauterine growth restriction (IUGR). When IUGR occurs at the threshold of neonate viability, accurate fetal weight estimation represents a valuable predictive factor to assess the probability of perinatal survival. Precise weight prediction before birth may minimize the perinatal morbidity and mortality associated with lower intrauterine growth.^1^

The normal development of the placenta, which occurs in the early stages of gestation, is dependent on the adequate invasion of trophoblastic cells in the decidual and myometrial segments of the spiral uterine arteries, which represent the most important source of irrigation in the uterine body region.^2^ ^3^ ^4^ Impaired trophoblast invasion (or abnormal placental implantation) is associated with elevated vascular resistance in the fetal-placental and uteroplacental circulation, and, consequently, with the development of preeclampsia and IUGR, a condition called placental insufficiency.^5^ ^6^

The reduction of fetal systemic blood flow due to fetal compensatory mechanisms in placental insufficiency leads to a decrease in the fetal growth rate.^7^ When these events lead to gestation in the second or early third trimesters, we call this early-onset placental insufficiency, and its severity is directly proportional to gestational age (GA), estimated fetal weight (EFW) and alterations found in Doppler mapping.^7^ Intrauterine growth restriction is a major consequence of placental insufficiency, and constitutes a significant public health problem, increasing the rates of neonatal morbidity and mortality and late postnatal consequences.^3^ ^4^ In general, this fetal pathology is a common clinical issue, present in 7 to 15% of all pregnancies.^8^

The perinatal outcome of fetuses affected by placental insufficiency is broadly dependent on the severity of the growth restriction, and EFW below the third percentile and/or abnormal findings in the umbilical artery (UA) represent the greatest risks for adverse perinatal results.^8^ ^9^ ^10^ ^11^ Other important prenatal determinants for the perinatal outcome are GA at birth and birth weight (BW), which is traditionally used as a predictive parameter of neonatal survival.^11^

Most EFW ultrasound formulae have been evaluated in multiple clinical conditions, but there is criticism of the indiscriminate use of these models in situations such as IUGR triggered by early-onset placental insufficiency. Only the Hadlock formula with four fetal biometric parameters (head circumference [HC], abdominal circumference [AC], femur length [FL] and biparietal diameter [BPD]) was tested in the population with altered Doppler velocimetry and high risk for IUGR.^12^

The present study aimed to assess the performance of 11 ultrasound formulae used to estimate fetal weight in premature fetuses with arterial and venous blood flow changes identified through Doppler velocimetry.

## Methods

Data were collected from a cohort of women and their fetuses submitted to ultrasound and Doppler velocimetry examinations who delivered at one of three maternity hospitals in the Rio de Janeiro metropolitan area: Instituto Fernandes Figueira, Clínica Perinatal Barra, and Clínica Perinatal Laranjeiras, between November 2002 and December 2013.

The inclusion criteria were: women on the 24th to 33rd weeks of pregnancy, calculated according to the date of the last menstrual period and confirmed through obstetric ultrasonography performed by the 20th week of pregnancy; presence of Doppler velocimetry levels compatible with fetal blood flow redistribution (increase of the pulsatility index [PI] of the UA above the 95th percentile for GA; presence of brain sparing reflex, with PI of the middle cerebral artery [MCA] below the 5th percentile for GA; AU with zero or reverse diastole in the UA); interval between the last ultrasound assessment of fetal biometry and birth not longer than five days; interval between last Doppler velocimetry exam and delivery not longer than 24 hours; and absence of signs of infection.

The exclusion criteria were multiple pregnancies, presence of fetal malformation assessed through prenatal care and/or confirmed on physical examination immediately after birth, and lack of reliable or available data to satisfy data collection.

Doppler velocimetry and ultrasonography were performed using the following devices: General Electric Voluson E6, and General Electric Voluson S6 (Boston, MA, US). All of the ultrasound examiners had at least two years of experience in obstetric examinations, and were certified by the Brazilian College of Radiology (CBR, in the Portuguese acronym) and the Brazilian Federation of Societies of Gynecology and Obstetrics (FEBRASGO, in the Portuguese acronym).

The fetal biometry measurements were BPD, HC, AC, and FL, which were based on previously described methodologies.^13^ ^14^ ^15^ The amniotic fluid index (AFI) was used to estimate the fluid volume, and was categorized as a dichotomous variable according to whether the values were normal or abnormal.^16^ Intrauterine growth restriction was defined in the present study as weight two standard deviations below the average BW for each GA.

The assessment of blood flow measurements of the resistance index (RI) and PI of the UA, MCA, and ductus venosus (DV) were obtained using a previously described methodology.^17^ ^18^ ^19^ Brain sparing reflex was considered when the PI of the MCA was below the 5th percentile for GA. The ratio between ventricular systole and atrial contraction (S/A ratio) on the DV was considered abnormal when the values exceeded 3.6, according to the local curve.^20^

Fetal weight was estimated with eleven different formulae using different numbers and combinations of BPD, HC, AC and FL obtained in the literature and available on the ultrasound equipment.

The EFW formulae listed were selected because they are widely used equations in the clinical practice, and because they are represented by fetal biometrics parameters available in routine ultrasound examinations. Formulae described for small fetuses, such as those of Mielke I and II, were not selected because they required the measurement of the transverse diameter of the abdomen, which is not part of the patterns used in the selected health units. The selected formulae are described in detail in Table 1.^21^ ^22^ ^23^ ^24^

**Table 1:** Formulae for fetal weight estimation

Formula	Parameters	Description
Campbell	AC	e^(–4.564 + 0.282 × AC-0.00331 × AC^2) [g,cm]
Hadlock I	BPD, HC, AC, FL	10^(1.3596 + 0.0064 × HC + 0.0424 × AC + 0.174 × FL + 0.00061 × BPD × AC-0.00386 × AC × FL) [g,cm]
Hadlock II	AC, FL	10^(1.304 + 0.05281 × AC + 0.1938 × FL-0.004 × AC × FL) [g,cm]
Hadlock III	BPD, AC, FL	10^(1.335–0.0034 × AC × FL + 0.0316 × BPD + 0.0457 × AC + 0.1623 × FL) [g,cm]
Hadlock IV	HC, AC, FL	10^(1.326–0.00326 × AC × FL + 0.0107 × HC + 0.0438 × AC + 0.158 × FL) [g,cm]
Hadlock V	BPD, AC	10^(1.1134 + 0.005845 × AC-0.000604 × AC^2–0.007365 × BPD^2 + 0.000595 × BPD × AC + 0.1694 × BPD) [g,cm]
Shepard	BPD, AC	10^(-1.7492 + 0.166 × BPD + 0.046 × AC-0.002546 × AC × BPD [kg,cm]
Warsof	BPD, AC	10^(-1.599 + 0.144 × BPD + 0.032 × AC-0.000111 × BPD^2 × AC) [kg,cm]
Weiner I	HC, AC, FL	10^(1.6961 + 0.02253 × HC + 0.01645 × AC + 0.06439 × FL) [g,cm]
Weiner II	HC, AC	10^(1.6575 + 0.04035 × HC + 0.01285 × AC) [g,cm]
Woo III	BPD, AC, FL	10^(1.54 + 0.15 × BPD + 0.00111 × AC^2–0.0000764 × BPD × AC^2 + 0.05 × FL-0.000992 × FL × AC [g,cm]

Abbreviations: AC, abdominal circumference; BPD, biparietal diameter; e, Euler number; FL, femur length; HC, head circumference.

Note: Source: Adapted from Abele et al (2010).^21^

The Hadlock formulae are often interchanged in previous studies, according to the understanding of the authors. In the present article, these equations were used according to the detailed description and numbering in Table 1. Following the birth, neonatologists immediately assisted the newborns. After initial care, birthweights were obtained and registered in scales of 5 g (Filizola, model BP Baby, São Paulo, SP, Brazil). The Local Ethics Committee approved this study under registration CAAE number 36546014.7.0000.5269.

The descriptive data analysis considered the mean and standard deviation. The formulae for fetal weight estimation listed in Table 1 were compared in terms of means for absolute percentage error (APE = |[Estimated weight – Birth weight] × 100/Birth weight|). All analyses were performed using the Statistical Package for the Social Sciences (SPSS, IBM Corp., Armonk, NY, US) software, version, 20, and the R (R Foundation for Statistical Computing, Vienna, Austria) software, version 2.15.1, with a significance level of 0.05 as reference.

A bivariate analysis was performed to compare the mean values for fetal weight estimation obtained with the different formulae with the following variables: growth pattern (appropriate for gestational age [AGA]/IUGR), GA at birth (< 28 weeks, 28–32 weeks, > 32 weeks), sex (male/female), AFI (normal/abnormal), UA status (normal/abnormal), and DV status (normal/abnormal). These analyses were performed to identify, for each of the equations used, which were related to the observed variations, considering a level of significance of 0.05.

## Results

In total, 194 patients met the research inclusion criteria. One case was excluded due to inconsistent registration of BPD in the medical record. All fetuses were delivered through cesarean section, except for the stillbirths, which were delivered vaginally. The descriptive analysis of the studied population is summarized in Table 2.

**Table 2:** Characteristics of the studied sample

Maternal age (years), mean ± SD	31 ± 6.2
Birth weight (grams), mean ± SD	918 ± 361.1
Gestational age at delivery (weeks), SD	28,8 ± 2.3
BW < 3rd percentile, frequency (percentage)	78 (40.2%)
Male sex frequency (percentage)	103 (53.1%)
Abnormal AFI frequency (percentage)	87 (44.8%)
Doppler UA AREDV frequency (percentage)	122 (62.9%)
Doppler DV abnormal S/A frequency (percentage)	60 (34.8%)
Stillborn frequency (percentage)	7 (3.6%)

Abbreviations: AFI, amniotic fluid index; AREDV, absent and reversed end-diastolic velocity; BW, birth weight; DV, ductus venosus; S/A, ventricular systole and atrial contraction ratio; SD, standard deviation; UA, umbilical artery.

The average fetal weight estimated through each formula is shown in Table 3.

**Table 3:** Representation of the 95% confidence interval of the mean estimated fetal weigth in grams using the eleven formulae

	n	Minimum	Maximum	Mean	Standard deviation
Campbell	194	282.31	3,312.29	981.6658	450.65413
Hadlock I	193	318.5	2,032.9	910.569	368.9429
Hadlock II	194	305.0	2,382.2	909.611	375.9173
Hadlock III	194	320.7	2,127.4	918.754	374.0832
Hadlock IV	193	317.7	2,042.4	909.641	366.9738
Hadlock V	194	345.4	2,246.0	983.673	409.3975
Shepard	194	373.8	2,264.5	1,008.021	419.2746
Warsof	194	359.6	1,966.5	920.546	370.5401
Weiner I	193	348.4	1,657.7	840.630	298.8425
Weiner II	193	312.6	1,862.8	883.734	307.7843
Woo III	194	375.2	2,092.7	949.585	385.2960

For APE, the formulae that demonstrated the best performance in the studied population were Hadlock I, II, III, and IV and Warsof, which had the lowest median values (Table 4).

**Table 4:** Performance of the eleven formulae for fetal weight estimation, expressed as the mean absolute percentage error (APE)

	n	Minimum	Maximum	Mean APE	Standard deviation
Campbell	194	0.01	99.54	13.8099	12.81752
Hadlock I	193	0.02	42.94	8.3193	7.32916
Hadlock II	194	0.05	47.86	9.4487	8.56320
Hadlock III	194	0.01	46.86	8.7386	7.69347
Hadlock IV	193	0.11	41.88	8.1745	7.23123
Hadlock V	194	0.02	48.81	11.5152	9.73483
Shepard	194	0.22	47.98	13.4033	10.14293
Warsof	194	0.00	45.49	9.3412	8.05797
Weiner I	193	0.05	33.24	9.7163	6.66733
Weiner II	193	0.01	37.21	9.5477	7.40668
Woo III	194	0.27	44.35	10.0275	8.15756

The Hadlock IV (HC, AC, FL), Hadlock I (BPD, HC, AC and FL), and Hadlock III (BDP, AC, FL) formulae had the lowest median APE values: 8.17, 8.32, and 8.74 respectively. A mean APE value < 10 was considered an indicator of satisfactory performance for each formula according to previous reports.

The cohort was divided into 2 groups per fetal growth: AGA, *n* = 116 (59.8%), and IUGR, *n* = 78 (40.2%). Intrauterine growth restriction was defined in the present study as weight two standard deviations below the average BW for each GA.

**Fig. 1:**
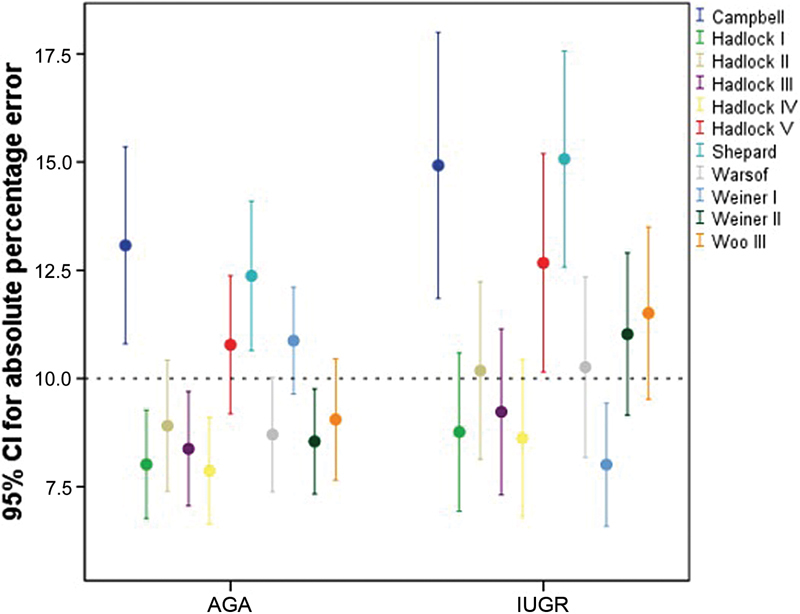
Graphical representation of the mean absolute percentage error for the 11 formulae and their respective 95% confidence intervals according to the pattern of fetal growth. Abbreviations: CI, confidence interval; APE, absolute percentage error; AGA, appropriate for gestational age; IUGR, intrauterine growth restriction.

Regarding the mean APE in AGA fetuses, Hadlock I, II, III, IV, and Warsof formulae demonstrated the best performance (8.02, 8.95, 8.41, 7.87, and 8.72 respectively). Hadlock IV (HC, AC, FL) had the lowest APE value. The same performance was observed in the IUGR fetus population, with a mean APE of 8.77, 10.18, 9.23, 8.62, and 10.26 respectively, as shown in Fig. 1. There was no significant difference between the median APE values of the AGA and IUGR groups, which were calculated using the Hadlock I (BPD, HC, AC and FL) and IV (HC, AC, FL) formulae.

The 11 formulae were also compared in groups defined per GA (24–28, 28–32 and 32–34 weeks), sex of the newborn (male or female), AFI (< 5 cm or ≥ 5 cm), UA Doppler status (PI > 95th percentile or absent/reverse diastole), and DV (normal or altered). The Hadlock formulae demonstrated no statistically significant difference in performance in the subgroup analysis per neonate sex, GA at birth, AFI, UA, or DV status (Table 5). The resulting table shows the existence or not of statistical association, considering a level of significance of 0.05.

**Table 5:** Influence of the clinical/ultrasonographic parameters on the average of the absolute percentage error for each formula

Formula	Growth pattern	GA at birth	Sex	AFI	UA status	DV status
Campbell	No	Yes	Yes	No	Yes	No
Hadlock I	No	No	No	No	No	No
Hadlock II	No	No	No	No	No	No
Hadlock III	No	No	No	No	No	No
Hadlock IV	No	No	No	No	No	No
Hadlock V	No	No	No	No	Yes	No
Shepard	No	No	No	No	Yes	No
Warsof	No	No	No	No	No	No
Weiner I	Yes	No	Yes	No	No	No
Weiner II	No	No	No	No	No	No
Woo III	No	No	No	No	No	No

Abbreviations: AFI, amniotic fluid index; DV, ductus venosus; GA, gestational age; UA, umbilical artery.

## Discussion

This study compared eleven formulae for fetal weight estimation in the clinical context of placental insufficiency observed on Doppler scan. This is a frequent pathology in pregnancy that is associated with an increased risk of growth restriction and low BW.^2^ ^3^ ^8^ ^11^ It is an original study in Brazil.

The formulae with the best performance for fetal weight estimation in the analyzed population were Hadlock I (BPD, HC, AC and FL) and IV (HC, AC and FL), which use four and three fetal biometric parameters respectively, based on the mean APE. This finding is consistent with what has already been documented in the medical literature for specific populations such as very premature fetuses and those with growth restriction.^21^ ^22^ ^24^ ^25^ ^26^ ^27^ ^28^ ^29^ ^30^ ^31^ ^32^ ^33^ ^34^ ^35^ The formulae with the poorest performance in our population were Campbell and Shepard.

The Hadlock formulae did not significantly differ in performance in the analyses according to fetal growth, fetal sex, GA at birth, AFI, UA, and DV status.^22^ This finding is similar to those obtained by studies that assessed cases of extreme prematurity for any reason and pregnancies with specific conditions such as preeclampsia.^21^ ^22^

The final fetal biometry measurement for the ultrasound weight estimation was obtained at least five days before birth. The last Doppler velocimetry measurement was performed in all patients no more than 24 hours before birth. This can be considered a particular strength of the study, because these parameters are more accurate than what is commonly found in the literature, which is a range of up to seven days between the last ultrasound examination and delivery.^21^ ^22^ ^25^ ^26^ Another quality that should be emphasized is the fact that the present study included a relatively large cohort, composed of 194 patients.

The study has some limitations that should be addressed. Only formulae based on BPD, HC, AC, and/or FL with circumferences measured by ellipsis were included; thus, the present findings cannot be extrapolated to formulae that use other parameters.^27^ We did not make adjustments for the interval between EFW and BW because this period was not longer than five days. This is a retrospective study; therefore, it carries a risk of loss of information due to flaws in medical records.

More studies, preferably with prospective designs and larger sample sizes, are necessary to corroborate the findings presented here, minimizing possible biases and enabling the extrapolation of the findings.

## Conclusion

The present study demonstrated the variability of performance among eleven different formulae for weight estimation in premature fetuses who experienced changes in blood flow. Our results indicate that the Hadlock formulae that use three (HC, AC and FL) or four (BPD, HC, AC and FL) biometric fetal parameters have the best results for this specific fetus population. Despite the reports of formulae designed specifically for premature and/or IUGR fetuses in the literature, the Hadlock I (BPD, HC, AC and FL) and IV (HC, AC and FL) formulae had fewer errors regarding BW in our study population. In addition, this better performance was not influenced by the clinical and ultrasound factors frequently present in early-onset placental insufficiency. Thus, considering the possible biases of this type of study design, our results indicate that the Hadlock I (BPD, HC, AC and FL) and IV (HC, AC and FL) formulae can be applied with satisfactory performance for fetal weight estimation in a population of fetuses with early-onset severe placental insufficiency.

## References

[1] Ricci A G Brizot MdeL Liao A W Nomura R M Zugaib M [Ultrasonographic accuracy of fetal weight estimation and influence of maternal and fetal factors] Rev Bras Ginecol Obstet 2011 33 09 240 245. Doi: 10.1590/S0100-72032011000900004 22189851

[2] Abuhamad A Z The role of Doppler ultrasound in obstetrics Philadelphia, PA Saunders Elsevier 2011 794 807

[3] Unterscheider J Daly S Geary M P, et al. Optimizing the definition of intrauterine growth restriction: the multicenter prospective PORTO Study Am J Obstet Gynecol 2013 208 04 2900 2.9E8. Doi: 10.1016/j.ajog.2013.02.007 10.1016/j.ajog.2013.02.007 23531326

[4] Nardozza L MM Araújo Junior E Vieira M F Rolo L C Moron A F Estimativa de peso ao nascimento utilizando a ultrassonografia bidimensional e tridimensional Rev Assoc Med Bras (1992) 2010 56 02 204 208. Doi: 10.1590/S0104-42302010000200020 20498996 10.1590/s0104-42302010000200020

[5] Baschat A A Doppler application in the delivery timing of the preterm growth-restricted fetus: another step in the right direction Ultrasound Obstet Gynecol 2004 23 02 111 118. Doi: 10.1002/uog.989 14770388 10.1002/uog.989

[6] Nelson D B Ziadie M S McIntire D D Rogers B B Leveno K J Placental pathology suggesting that preeclampsia is more than one disease Am J Obstet Gynecol 2014 210 01 660 6.6E8. Doi: 10.1016/j.ajog.2013.09.010 10.1016/j.ajog.2013.09.010 24036400

[7] Baschat A A Gembruch U Harman C R The sequence of changes in Doppler and biophysical parameters as severe fetal growth restriction worsens Ultrasound Obstet Gynecol 2001 18 06 571 577. Doi: 10.1046/j.0960-7692.2001.00591.x 11844191 10.1046/j.0960-7692.2001.00591.x

[8] Moreira Neto A R Córdoba J CM Peraçoli J C Etiologia da restrição do crescimento intrauterino (RCIU) Comun Ciênc Saúde 2011 22 21 30

[9] Robson S C Martin W L Morris R K The investigation and management of the small-for-gestational-age fetus London RCOG 2013

[10] Seravalli V Baschat A A A uniform management approach to optimize outcome in fetal growth restriction Obstet Gynecol Clin North Am 2015 42 02 275 288. Doi: 10.1016/j.ogc.2015.01.005 26002166 10.1016/j.ogc.2015.01.005

[11] Unterscheider J O'Donoghue K Daly S, et al. Fetal growth restriction and the risk of perinatal mortality-case studies from the multicentre PORTO study BMC Pregnancy Childbirth 2014 14 63. Doi: 10.1186/1471-2393-14-63 24517273 10.1186/1471-2393-14-63 PMC3923738

[12] Carvalho P RN Sá R AM Gomes S C Jr Lopes L M Moreira M EL Evaluation of Hadlock's formula in premature fetuses with severe Doppler abnormalities J Perinat Med 2011 39 1 5

[13] Campbell S Wilkin D Ultrasonic measurement of fetal abdomen circumference in the estimation of fetal weight Br J Obstet Gynaecol 1975 82 09 689 697. Doi: 10.1111/j.1471-0528.1975.tb00708.x 1101942 10.1111/j.1471-0528.1975.tb00708.x

[14] O'Brien G D Queenan J T Campbell S Assessment of gestational age in the second trimester by real-time ultrasound measurement of the femur length Am J Obstet Gynecol 1981 139 05 540 545. Doi: 10.1016/0002-9378(81)90514-7 7193418 10.1016/0002-9378(81)90514-7

[15] Shepard M Filly R A A standardized plane for biparietal diameter measurement J Ultrasound Med 1982 1 04 145 150. Doi: 10.7863/jum.1982.1.4.145 6152944 10.7863/jum.1982.1.4.145

[16] Phelan J P Smith C V Broussard P Small M Amniotic fluid volume assessment with the four-quadrant technique at 36-42 weeks' gestation J Reprod Med 1987 32 07 540 542 3305930

[17] Arduini D Rizzo G Normal values of Pulsatility Index from fetal vessels: a cross-sectional study on 1556 healthy fetuses J Perinat Med 1990 18 03 165 172. Doi: 10.1515/jpme.1990.18.3.165 2200862 10.1515/jpme.1990.18.3.165

[18] Rizzo G Capponi A Talone P E Arduini D Romanini C Doppler indices from inferior vena cava and ductus venosus in predicting pH and oxygen tension in umbilical blood at cordocentesis in growth-retarded fetuses Ultrasound Obstet Gynecol 1996 7 06 401 410. Doi: 10.1046/j.1469-0705.1996.07060401.x 8807755 10.1046/j.1469-0705.1996.07060401.x

[19] Wladimiroff J W Tonge H M Stewart P A Doppler ultrasound assessment of cerebral blood flow in the human fetus Br J Obstet Gynaecol 1986 93 05 471 475. Doi: 10.1111/j.1471-0528.1986.tb08656.x 3518788

[20] Sá R AM Chaves Netto H Amim J Jr, et al. Ductus venosus velocimetry in normal pregnancy Int J Gynaecol Obstet 2000 70(S1):A28. Doi: 10.1016/S0020-7292(00)82042-1

[21] Abele H Hoopmann M Wagner N Hahn M Wallwiener D Kagan K O Accuracy of sonographic fetal weight estimation of fetuses with a birth weight of 1500 g or less Eur J Obstet Gynecol Reprod Biol 2010 153 02 131 137. Doi: 10.1016/j.ejogrb.2010.07.007 20675035 10.1016/j.ejogrb.2010.07.007

[22] Geerts L Widmer T Which is the most accurate formula to estimate fetal weight in women with severe preterm preeclampsia? J Matern Fetal Neonatal Med 2011 24 02 271 279. Doi: 10.3109/14767058.2010.485232 21231823 10.3109/14767058.2010.485232

[23] Woo J S Wan C W Cho K M Computer-assisted evaluation of ultrasonic fetal weight prediction using multiple regression equations with and without the fetal femur length J Ultrasound Med 1985 4 02 65 67 3882988 10.7863/jum.1985.4.2.65

[24] Blumenfeld Y J Lee H C Pullen K M Wong A E Pettit K Taslimi M M Ultrasound estimation of fetal weight in small for gestational age pregnancies J Matern Fetal Neonatal Med 2010 23 08 790 793. Doi: 10.3109/14767050903387052 19968588 10.3109/14767050903387052

[25] Hadlock F P Harrist R B Sharman R S Deter R L Park S K Estimation of fetal weight with the use of head, body, and femur measurements--a prospective study Am J Obstet Gynecol 1985 151 03 333 337. Doi: 10.1016/0002-9378(85)90298-4 3881966 10.1016/0002-9378(85)90298-4

[26] Anderson N G Jolley I J Wells J E Sonographic estimation of fetal weight: comparison of bias, precision and consistency using 12 different formulae Ultrasound Obstet Gynecol 2007 30 02 173 179 17557378 10.1002/uog.4037

[27] Smulian J C Ranzini A C Ananth C V Rosenberg J C Vintzileos A M Comparison of three sonographic circumference measurement techniques to predict birth weight Obstet Gynecol 1999 93(5 Pt 1):692 696. Doi: 10.1016/S0029-7844(98)00517-1 10912969 10.1016/s0029-7844(98)00517-1

[28] Dudley N J A systematic review of the ultrasound estimation of fetal weight Ultrasound Obstet Gynecol 2005 25 01 80 89. Doi: 10.1002/uog.1751 15505877 10.1002/uog.1751

[29] Kurmanavicius J Burkhardt T Wisser J Huch R Ultrasonographic fetal weight estimation: accuracy of formulas and accuracy of examiners by birth weight from 500 to 5000 g J Perinat Med 2004 32 02 155 161. Doi: 10.1515/JPM.2004.028 15085892 10.1515/JPM.2004.028

[30] Medchill M T Peterson C M Kreinick C Garbaciak J Prediction of estimated fetal weight in extremely low birth weight neonates (500-1000 g) Obstet Gynecol 1991 78 02 286 290 2067777

[31] Burd I Srinivas S Paré E Dharan V Wang E Is sonographic assessment of fetal weight influenced by formula selection? J Ultrasound Med 2009 28 08 1019 1024. Doi: 10.7863/jum.2009.28.8.1019 19643784 10.7863/jum.2009.28.8.1019

[32] Jouannic J M Grangé G Goffinet F Benachi A Carbrol D Validity of sonographic formulas for estimating fetal weight below 1,250 g: a series of 119 cases Fetal Diagn Ther 2001 16 04 254 258. Doi: 10.1159/000053923 11433125 10.1159/000053923

[33] Shamley K T Landon M B Accuracy and modifying factors for ultrasonographic determination of fetal weight at term Obstet Gynecol 1994 84 06 926 930 7970471

[34] Siemer J Egger N Hart N, et al. Fetal weight estimation by ultrasound: comparison of 11 different formulae and examiners with differing skill levels Ultraschall Med 2008 29 02 159 164. Doi: 10.1055/s-2007-963165 17602369 10.1055/s-2007-963165

[35] Townsend R R Filly R A Callen P W Laros R K Factors affecting prenatal sonographic estimation of weight in extremely low birthweight infants J Ultrasound Med 1988 7 04 183 187. Doi: 10.7863/jum.1988.7.4.183 3285021 10.7863/jum.1988.7.4.183

